# Isometric muscle strength profile of lower limbs for children and adolescents between 7 and 15 years of age

**DOI:** 10.7717/peerj.20799

**Published:** 2026-02-23

**Authors:** Oscar Bustos-Moyano, Eduardo Guzmán-Muñoz, Pablo Valdes-Badilla, Diego Frugone-Zaror, Flor Miño-Cornejo, Felipe Araya-Quintanilla, Guillermo Mendez-Rebolledo

**Affiliations:** 1 Departamento de Kinesiología, Facultad de Ciencias de la Salud, Universidad Católica del Maule, Talca, Región del Maule, Chile; 2 Laboratorio de Investigación Somatosensorial y Motora, Escuela de Kinesiología, Facultad de Salud, Universidad Santo Tomas, Talca, Región del Maule, Chile; 3 Departamento de Ciencias de la Actividad Física, Facultad de Ciencias de la Educación, Universidad Católica del Maule, Talca, Chile; 4 Sports Coach Career, School of Education, Universidad de Viña del Mar, Viña del Mar, Región de Valparaíso, Chile; 5 Escuela de Educación Física, Facultad de Educación, Universidad Autónoma de Chile, Talca, Chile; 6 Escuela de Kinesiología, Facultad de Odontología y Ciencias de la Rehabilitación, Universidad San Sebastián, Santiago, Región Metropolitana, Chile

**Keywords:** Isometric strength, Lower limb, Children, Adolescents, Musculoskeletal development

## Abstract

**Background:**

Muscle strength is a key determinant of health-related physical fitness and has become a significant biological predictor of overall health and lifespan. This study aimed to characterize the development of isometric strength in the lower limbs of Chilean schoolchildren and adolescents aged 7 to 15 years. Specifically, it sought to: (a) determine when sex-based differences in lower limb strength first appear; (b) identify key developmental stages where significant gains in muscle strength occur in boys and girls; and (c) analyze the contribution of individual muscle groups to total lower limb strength while accounting for sex and age differences.

**Methodology:**

This cross-sectional study evaluated the maximum isometric strength of seven lower limb muscle groups in a sample of 302 Chilean children, divided into nine age groups at one-year intervals. Hand-held dynamometry was used for isometric strength assessment. Data analysis included a two-factor analysis of variance (ANOVA) for maximum isometric strength and a stepwise multiple linear regression analysis to the entire sample.

**Results:**

Multiple comparisons showed significant differences between the ages of 9 and 12; and sex. The narrowest age range in the progression of maximum isometric strength were: 9–11 years for knee flexors in females (*p* = 0.0201) and 9–12 years for males (*p* = 0.0008). Hip flexors, dorsiflexors, hip extensors and knee extensors explained the highest percentage of variance (*R*^2^ = 0.897, *p* < 0.0001) in the total lower limb strength.

**Conclusion:**

Our findings show that lower limb isometric strength in Chilean schoolchildren and adolescents increases from age 10, with boys outperforming girls by age 15. Hip flexors mainly explain total strength, offering a useful reference to detect within and between subject strength deficits.

## Introduction

Muscle strength is defined as the force generating capacity during voluntary contraction, a foundational concept in exercise physiology (Jones & Stratton, 2000). Muscle strength is a key factor in physical fitness and serves as an indicator of overall health and longevity (Hébert et al., 2015; Orsso et al., 2019). A reduction in both muscle mass and strength—occurring throughout development and aging—has been linked to various negative health consequences (Orsso et al., 2019). Low muscle mass and strength in youth are linked to higher incidences of metabolic and cardiovascular disorders, musculoskeletal injuries, and neurodevelopmental challenges (Peterson et al., 2016; Muehlbauer, Gollhofer & Granacher, 2015; Read et al., 2018; Delezie & Handschin, 2018), finally, recent studies show also a risk of developing pediatric dynapenia that could be reversed by resistance training (Ruas et al., 2024). For these reasons, a proper assessment of muscle strength is important in pediatric musculoskeletal, cardiovascular and neurological rehabilitation (Daloia et al., 2018; Hébert et al., 2015; Muehlbauer, Gollhofer & Granacher, 2015).

For a proper muscle strength assessment in a young population, there are various techniques available, like isokinetic dynamometry, hand-held dynamometry as well as clinical and field tests (Jones & Stratton, 2000). To choose the suitable assessment it must take into account expensiveness, special requirements (training and adaptation), reliability and validity of the test and evaluator (Cuthbert & Goodheart Jr, 2007). Given its reliability in measuring isolated joint strength, hand-held dynamometry (HHD) was selected as the primary assessment tool (Jones & Stratton, 2000). In this context, isometric strength refers to the generation of muscle tension without any accompanying change in muscle length or joint angle (Komi, 1984). The use on HHD provide the maximum (peak) isometric strength during the performance of a standardized test for the majority of clinically significant muscle groups in children and adolescents (Jones & Stratton, 2000; Schrama et al., 2014), demonstrating a high reliability and validity (Daloia et al., 2018; Davis et al., 2017; Hébert et al., 2011; Van den Beld et al., 2006).

Several studies have examined the relationship between isometric strength in age, sex, weight, and height in children and adolescents using both absolute (Newtons) or relative values (N/kg) for muscle strength (Hébert et al., 2011; Orsso et al., 2019; Rauch et al., 2002; Schrama et al., 2014). According to these studies, it has been observed that there are no significant differences in strength development between boys and girls before the age 10. However, around the ages of 11–12, boys tend to develop greater strength than girls (Daloia et al., 2018; Escobar, Munoz & Dominguez, 2017; Hébert et al., 2011; Mendez-Rebolledo et al., 2022). For example, it has been observed that the wrist flexors and elbow flexors muscles undergo greater development of muscular strength between the ages of 11 and 15 in males, resulting in differences in strength compared to females. Furthermore, during early adolescence, youths experience significant strength development in some muscle groups (Mendez-Rebolledo et al., 2022). Specifically, the shoulder flexor muscles have been observed to account for approximately 74% of the total strength generated in the upper body (Mendez-Rebolledo et al., 2022). These findings provide valuable guidance for clinicians and coaches in evaluating and designing strength training programs tailored to specific youth developmental periods. Nevertheless, the isometric strength profile and the influence of each muscle group in the total strength and functionality of lower limb between the ages of 7 and 15 remain unknown, particularly considering the significant changes in muscle mass and bone length experienced during puberty. In addition to morphological and hormonal differences, neuromuscular factors also contribute to strength development across childhood and adolescence. Specifically, pre-pubertal children have been shown to have a reduced ability to voluntarily activate their motor units compared to post-pubertal adolescents and adults. Studies using the interpolated twitch technique (ITT) have demonstrated that voluntary activation in children can be 15–30% lower than in adults (Dotan et al., 2012; Stackhouse, Binder-Macleod & Lee, 2005). This incomplete activation is attributed to immaturity of the central nervous system and motor pathways, and it may partially explain the lower isometric strength observed in younger age groups, regardless of muscle size. On the other hand, while chronological age is commonly used in pediatric research, it does not always reflect an individual’s biological maturation status. Several studies have shown that variability in muscle strength among children of the same chronological age can be partially explained by differences in maturity status (Malina, Bouchard & Bar-Or, 2004; Lloyd & Oliver, 2012). Biological maturity, often assessed *via* Tanner staging or peak height velocity (PHV), is a more precise indicator of neuromuscular development and strength potential. In the present study, we acknowledge this limitation and suggest that future research should incorporate biological maturity assessments to better understand strength progression during youth. Adequate strength is vital for the development of fundamental motor skills such as walking, running, and jumping (Macfarlane, Larson & Stiller, 2008). For these reasons, the purpose of this study was to determine the isometric strength profile of lower limb muscles of children and adolescents between 7 to 15 years of age. The hip, knee and ankle muscle groups were evaluated with the HHD method. The secondary objectives of this study are (a) to determine the statistically significant age at which sex-related differences in lower limb isometric strength become evident; (b) to determine the age range during which significant progression of isometric strength is observed in girls and boys; and (c) to examine the role of each muscle group’s isometric strength on the total lower limb strength in the study sample, considering sex and age as descriptive covariates rather than adjustment factors.

## Materials and Methods

### Study design

A cross-sectional study was conducted following the Strengthening the Reporting of Observational Studies in Epidemiology (STROBE) guidelines (von Elm et al., 2007), ensuring methodological rigor and consistency. The research complied with all the relevant national regulations and institutional policies, including the Declaration of Helsinki, and was approved by the ethics committee of the Universidad Santo Tomás, Chile (Folio ID-98-19). All participants and their parents/guardians were fully informed about the study procedures. Written informed consent was obtained from parents/guardians, and written assent was obtained from participants prior to data collection.

### Participants

The sample size was calculated based on Hébert et al. (2015), using an effect size (S) of 0.27 and a precision level (W) of 0.38 with a 95% confidence interval (Zα = 1.96) as shown in the formula:

\begin{eqnarray*}Sample~Size= \frac{4z\alpha \wedge 2~S\wedge 2}{{W}^{2}} , \end{eqnarray*}

which resulted in a minimum requirement of seven participants per sex per age group. This calculation allowed us to detect significant differences in normalized isometric strength values. Additionally, our subgroup sizes are in line with other studies using HHD to assess pediatric strength across various world regions, including Canada (Hébert et al., 2015), Brazil (Daloia et al., 2018), and Chile (Escobar, Munoz & Dominguez, 2017). Children were recruited geographically from local schools of the central area of Chile, between the ages of 7 to 15 years. Participants of the study had typical development and were Spanish speaking Latino ethnic. Ethnicity was reported following the Standards of the Classification of Federal Data on Race and Ethnicity (White House, 1997). Inclusion criteria for participants demanded intact cognitive functions to understand the orders given by the evaluator. Exclusion of the participants were if they presented (a) pain during assessment and procedures, (b) a history of medical, neurological, or musculoskeletal impairments that could affect muscle strength measurements, (c) previous surgeries of the lower limbs and/or spine, or participation in competitive sports during or six months prior to the measurements.

### Measures

Body weight and height were measured using a Seca model 220 stadiometer (Hamburg, Germany), ensuring a precision of 0.1 kg of weight and 0.1 cm for height. Measurements of the isometric muscle strength were performed on the dominant lower limb; this was determined by asking the participant to kick a ball comfortably. If there was discrepancy between the test results, participants were asked to indicate their preferred leg. The maximum isometric muscle strength of the hip flexors, hip extensors and hip abductors, as well as knee flexors and knee extensor muscles, and ankle dorsiflexors and plantar flexors muscle group were assessed with a calibrated HHD (MMT 01165, Lafayette Manual Muscle Test System, Lafayette, IN, USA). Intra-rater reliability was assessed using intraclass correlation coefficients (ICC = 0.77–0.95) and the standard error of measurement (SEM = 0.06–0.08 N/kg). Although absolute agreement metrics such as Bland–Altman limits were not included, this decision was based on recent methodological literature and pediatric studies using HHD. For instance, Jorquera-Cáceres et al. (2024) showed that SEM offers sufficient insight into measurement precision for normative studies, particularly when combined with rigorous evaluator training and protocol standardization. As our study focused on group-level isometric strength profiling rather than individual change over time, we considered the ICC-SEM combination appropriate and methodologically robust. The sequence of the muscle evaluation was organized—including the standardized test position and the specific placement of the hand-held dynamometer—by adapting protocols from previous studies (Daloia et al., 2018; Escobar, Munoz & Dominguez, 2017; Hébert et al., 2011), as detailed in Table 1.

**Table 1:** Order of muscles assess, standardized positions, and hand-held dynamometer placement for each muscle group.

**Muscle group**	**Participan position**	**Dynamometer position**	**Image of dynamometer position**
**Hip Flexors**	Supine position on a stretcher 90° hip flexion, 90° knee flexion and leg on top of box	Perpendicular, on the distal third part of the anterior surface of the thigh, on the superior edge of the patella.	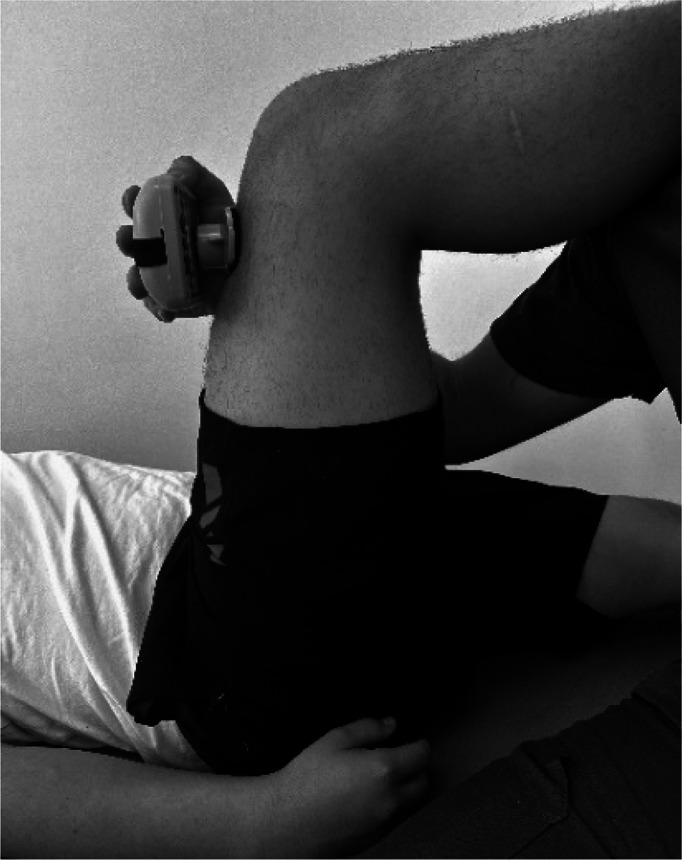
**Hip Extensors**	Supine position on a stretcher 90° hip flexion, 90° knee flexion and leg on top of box	Perpendicular, on the distal third part of the posterior surface of the thigh, proximal to popliteal skinfold.	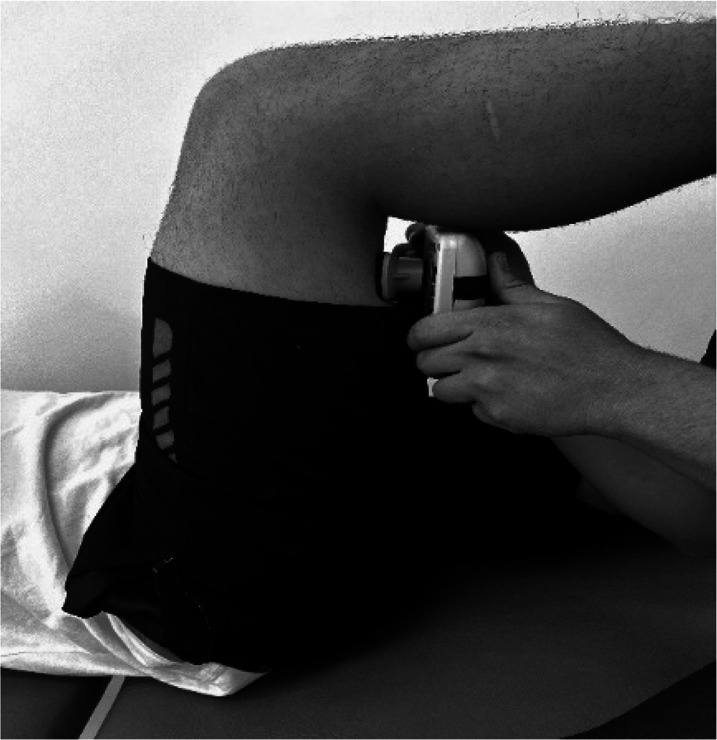
**Hip Abductors**	Supine position on a stretcher 0° hip flexion, 0° knee flexion and contralateral thigh fixed and stabilized on the distal third part.	Perpendicular, distal third part of the thigh, on the lateral condyle of the femur	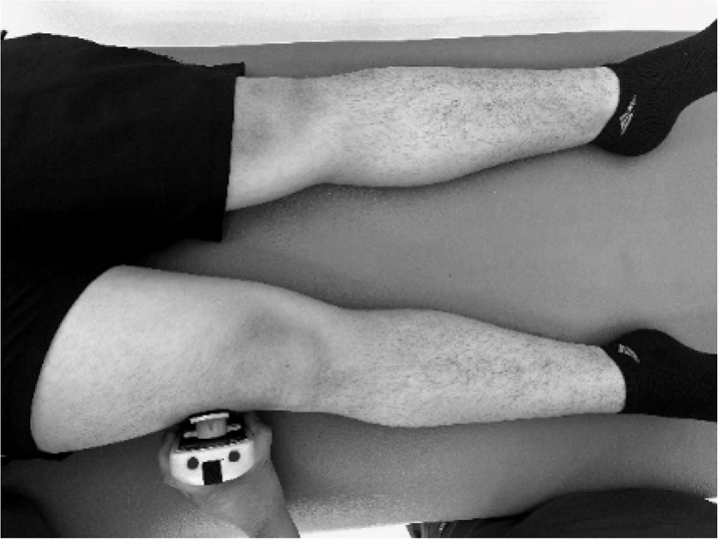
**Knee Flexors**	Sitting neutral position with feet on the ground. 90° hip flexion, 90° knee flexion and trunk upright without support.	Perpendicular, distal third of the posterior surface of the lower leg, proximal to the ankle	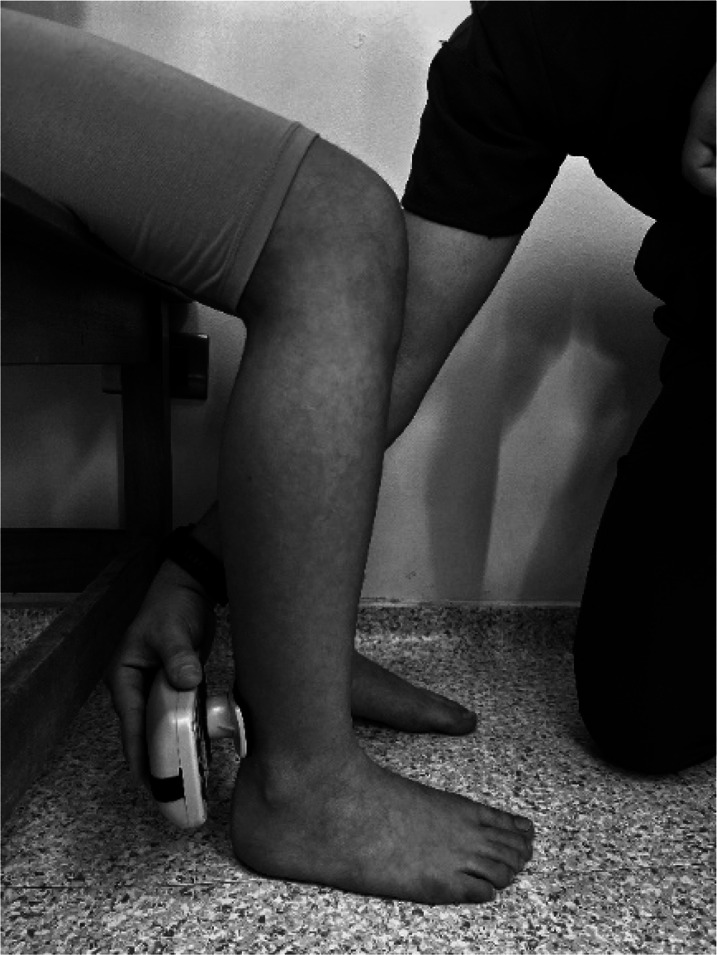
**Knee Extensor**	Sitting neutral position with feet on the ground. 90° hip flexion, 90° knee flexion and trunk upright without support.	Perpendicular, distal third of the anterior surface of the lower leg, proximal to the ankle	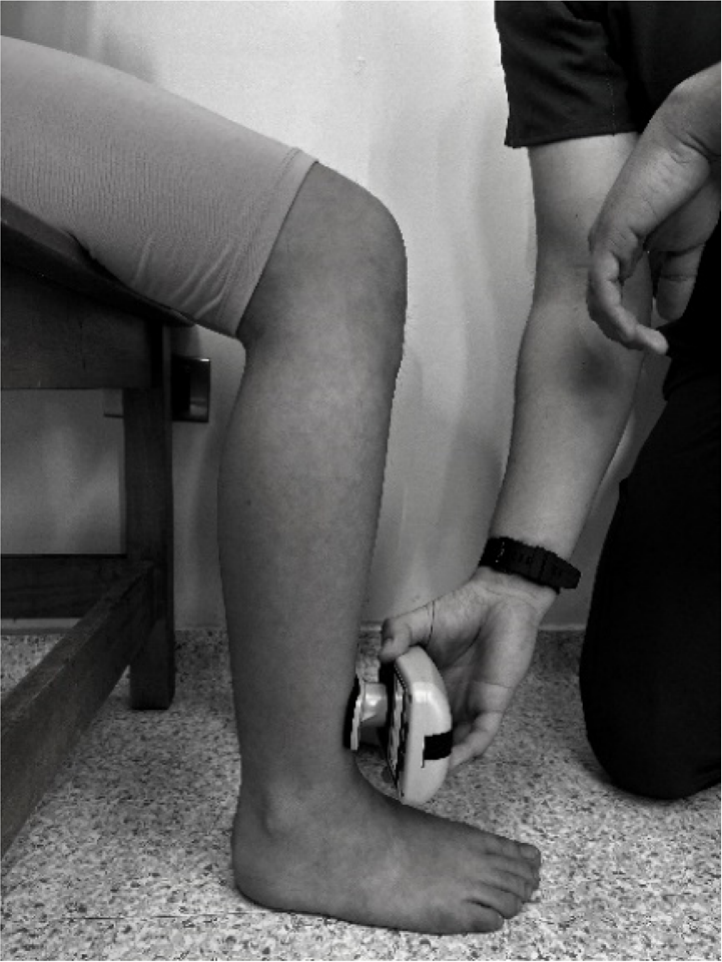
**Ankle Dorsiflexors**	Supine position on a stretcher 0° hip flexion, 0° knee flexion, and ankle in neutral position on edge of stretcher	Perpendicular, dorsal surface of the foot, proximal to the metatarsophalangeal joint	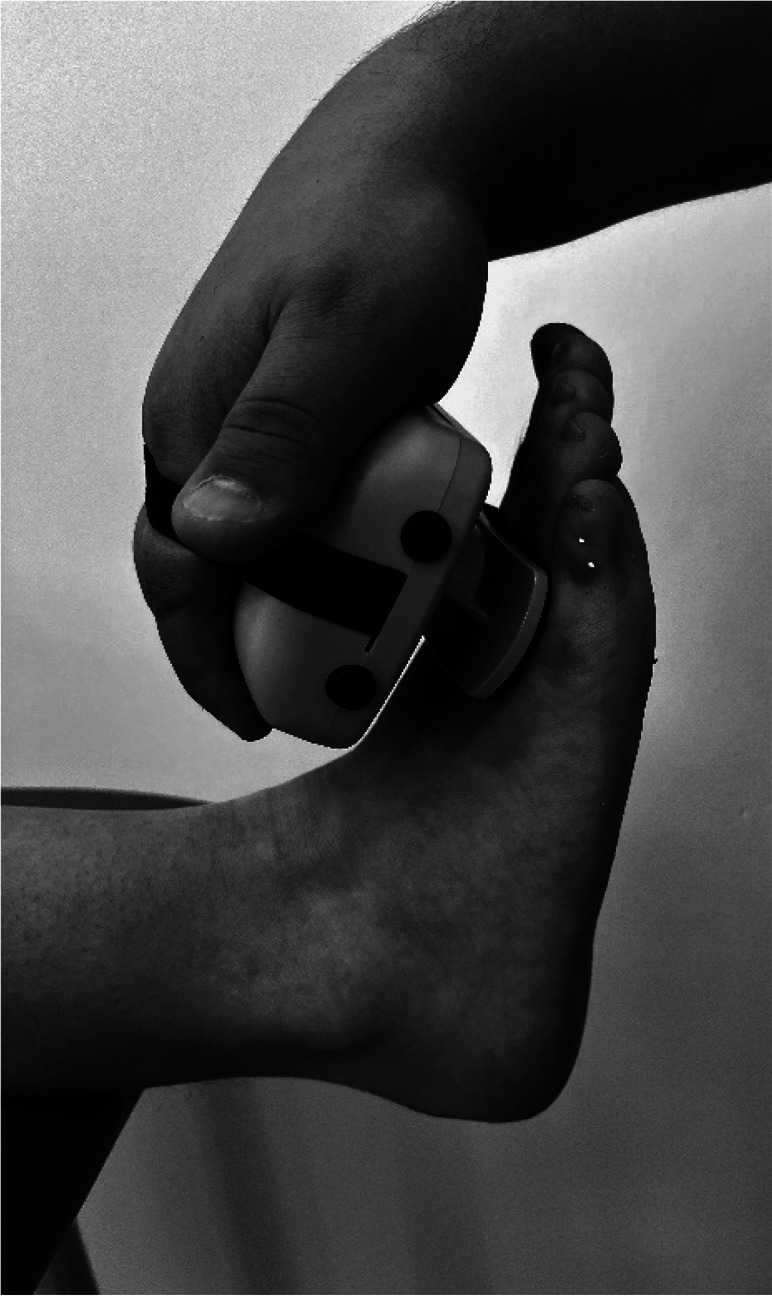
**Ankle Plantarflexors**	Supine position on a stretcher 0° hip flexion, 0° knee flexion, and ankle in neutral position on edge of stretcher	Perpendicular, plantar surface of the foot, proximal to the metatarsophalangeal joint	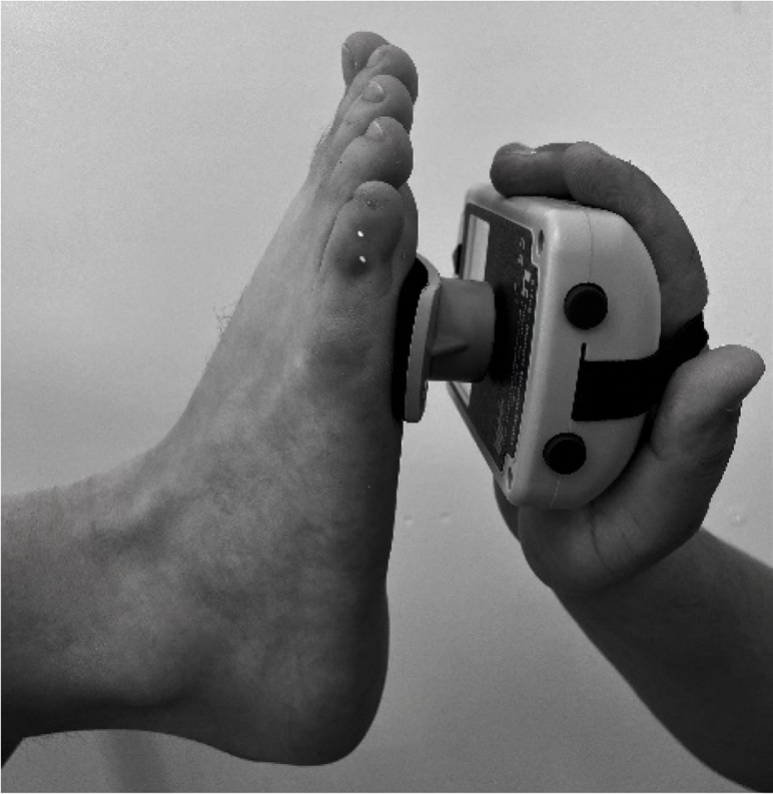

## Anthropometric Assessment

To improve comparability across age groups, BMI Z-scores adjusted for age and sex were calculated using the World Health Organization (2007) growth reference standards for children and adolescents aged 5 to 19 years (boys and girls), as presented in the Chilean Ministry of Health nutritional evaluation tables (Ministerio de Salud de Chile, 2018). The Z-scores were computed using the LMS method based on age in months and sex, allowing for standardized comparisons across developmental stages.

The main researcher provided twelve hours of training to an evaluator, who was a physical therapist, on all dynamometry procedures. Participants were instructed to wear sports clothes and be barefoot. All measurements were conducted in a single testing session, with a duration of one hour per participant. A “make test”—method where a child exerts maximum force against a resistance, held constant for a specific duration- was employed to measure the maximal isometric strength of lower limb muscle. In this protocol the evaluator held the dynamometer fixed while the subject applied maximum force (Jones & Stratton, 2000; Schmidt et al., 2013; Stratford & Balsor, 1994). Participants were instructed to gradually increase their effort until reaching maximum force during each isometric contraction receiving consistent verbal encouragement (*e.g.*, “harder, harder, harder”) to ensure maximal exertion during each trial. This approach was selected to ensure safety and control during testing, particularly in younger children, and to avoid excessive joint stress or compensatory movements. While some protocols suggest encouraging participants to ‘push as hard and as fast as possible’ to enhance peak force output, we opted for a controlled buildup of force to prioritize measurement consistency and participant comfort in this pediatric population. Each subject performed three trials per muscle group, and the mean value of these trials was calculated to represent their maximal isometric strength. The average of the three attempts was used for subsequent analysis. Prior to the assessment, a submaximal contraction attempt was performed for each muscle group. This preliminary attempt served multiple purposes, including ensuring the participant’s understanding of the task, confirming joint stability, and serving as a warm-up (Hébert et al., 2015). The contraction (attempt) that each participant did was progressive and was held for 5-s, followed by a 30-s rest period that minimized the effects of fatigue. To account for individual differences in body size, peak isometric strength values were normalized by dividing by body mass (N/kg). This approach has been commonly used in pediatric HHD studies (Hébert et al., 2015; Daloia et al., 2018; Escobar, Munoz & Dominguez, 2017). However, we acknowledge that this method does not control for differences in lean body mass or biological maturation, which may affect the interpretation of relative strength, especially in small muscle groups such as the dorsiflexors or in adolescents undergoing pubertal growth. By normalizing the data, it was assumed that there would be no significant statistical differences in anthropometric measurements within a specific age group. This methodology guarantees that the recorded isometric muscle strength data accurately represent the given age and sex categories, facilitating meaningful comparisons between individuals (Mattiello-Sverzut, 2020). The total lower limb was calculated by adding a normalized maximum isometric strength of the hip flexors, hip extensors, hip abductors, knee extensors, knee flexors, and dorsiflexors and plantarflexors muscles.

### Statistical analysis

Data were analyzed using IBM SPSS Statistics ver. 25.0 (IBM Co., Armonk, NY, USA). A significance level (alpha level) of <0.05 was used for all tests. Grouped by age and sex, means and standard deviation were calculated for the maximum isometric muscle strength of the lower limb in children and adolescents. Outlays were considered and removed when their values were >3 standard deviations from the mean. Additionally, normality distribution of the data was assessed by the Shapiro–Wilk test (Hébert et al., 2015).

A total of seven two-way ANOVAs (age × sex) were performed, one for each lower limb muscle group (hip flexors, hip extensors, hip abductors, knee flexors, knee extensors, plantar flexors, and dorsiflexors), to examine main effects and interactions across age and sex. When significant main or interaction effects were found, Bonferroni-corrected post hoc comparisons were conducted to identify specific age intervals showing significant differences in normalized isometric strength values. These intervals are described in the results as ’periods of significant isometric strength progression’, rather than developmental milestones, to avoid conflating chronological with biological maturation. To determine the narrowest age range during which significant strength progression occurred, Bonferroni-corrected post hoc pairwise comparisons were performed between all consecutive age groups. The smallest interval showing a statistically significant difference (*p* < 0.05) in mean normalized strength was recorded for each muscle group. To examine the effect size an Eta-squared (η^2^) for ANOVA was used where less than 0.06 was classified as “small”, 0.07–0.14 as “moderate”, and greater than 0.14 as “large”. Furthermore, Cohen *d* for paired samples was used as an indicator of the effect size, where less than 0.2 was classified as “trivial”, 0.2–0.5 as “small”, 0.5–0.8 as “moderate”, and greater than 0.8 as “large” (Cohen, 1992).

Finally, to identify the role of the isometric strength of each muscle group on the total muscle strength of the lower limb, a relationship between the maximum isometric strength of each muscle group and the total muscle strength of lower limb was analyzed using Pearson correlation test, where a correlation coefficient (*r*) from 0–0.4 was considered as “weak”, 0.41–0.7 as “moderate”, and 0.71–1.0 as “strong”. To examine the contribution of individual muscle groups to total lower limb isometric strength (defined as the sum of normalized isometric strength values), we performed multiple linear regression analysis on the entire sample to explore the relative contribution of each muscle group to total lower limb strength. Prior to modeling, collinearity diagnostics were conducted. Tolerance values > 0.10 and variance inflation factors (VIFs) < 5 were considered acceptable to confirm low multicollinearity (O’brien, 2007). Variables exceeding these thresholds were excluded from the final model. To interpret the contribution of each predictor, we examined standardized beta coefficients and changes in explained variance (Δ*R*^2^). While we recognize that Δ*R*^2^ values depend on the order of entry of variables, this approach was selected based on methodological guidelines for exploratory regression models (Krasikova, LeBreton & Tonidandel, 2011). Results should therefore be interpreted as relative contributions rather than definitive causal weights.

## Results

**Figure 1:**
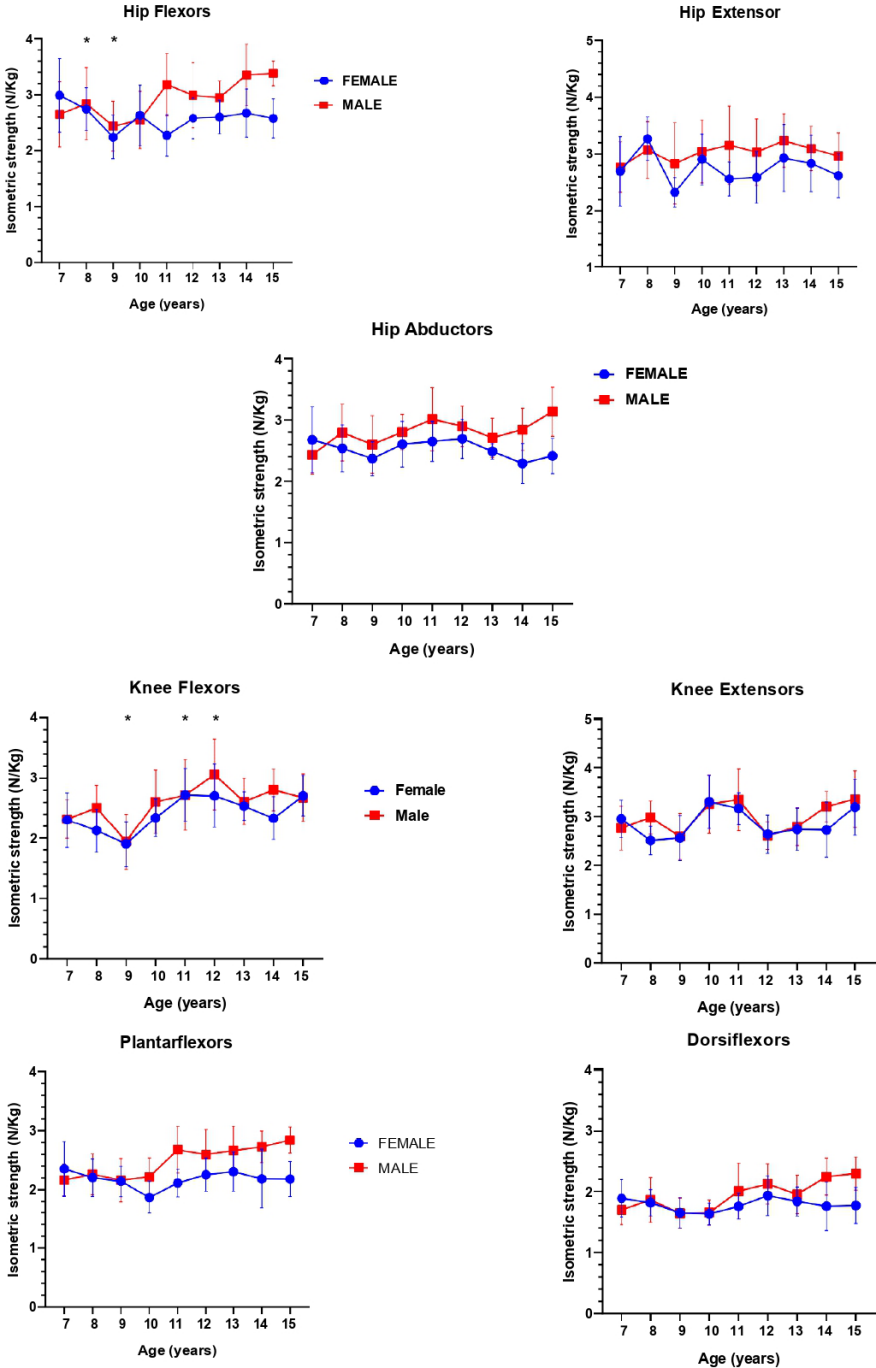
A linear development of maximum isometric muscle strength for boy and girls * *P* < 0, 05, significant differences between girls and boys.

A total of 302 individuals (50,3% female) were included in the analysis. Figure 1 summarizes the maximum isometric muscle strength by means of superimposed symbols (at the mean) for each muscle group, age and sex. Table 2 shows the number of participants grouped by age and average body mass (kg), height (cm), Z-score BMI and physical activity questionnaire for older children (PAQ-A/C).

Furthermore, the intrarater reliability of the maximal isometric muscle strength assessments was excellent (ICC = 0.77–0.95; SEM = 0.06–0.08 N/kg). Detailed ICC and SEM values for each muscle group across age categories are presented in Table 3. There was a significant difference in the boy and girls’ height at the age of 14 years and significant difference in body mass at the age of 15 years, but no differences in body mass index were observed for any age group.

The data of maximum isometric strength for all muscle groups (Table 3) were normally distributed. The two-way ANOVA revealed a significant interaction between age and sex for hip flexors (F(8) = 2.306, *p* = 0.0211, η^2^ = 0.06 “small”) and plantar flexors (F(8) = 2.817, *p* = 0.0052, η^2^ = 0.06 “small”), both small effects. Bonferroni-adjusted multiple comparisons showed that the narrowest intervals of significant isometric strength gains occurred: between 9–11 years for knee flexors in females (*p* = 0.0201, *d* = 1.03), 9–12 years for knee flexors in males (*p* = 0.0008, *d* = 1.20), and 8–9 years for hip extensors in females (*p* = 0.0280, *d* = 1.52). These intervals are depicted in Fig. 1, where asterisks indicate statistically significant age differences in each muscle group by sex.

The total isometric muscle strength of the lower limb showed a moderate significant correlation with all muscle groups (*r* = 0.623–0.738, *p* = 0.0001). Instead, total isometric muscle strength of the lower limb did not show significant correlation with age (*r* = 0.197, *p* = 0.001), however it did in height (*r* = 097, *p* = 0.097). The stepwise multiple linear regression model was performed on the entire sample to explore the relative contribution of each muscle group to total lower limb strength. Although sex and age were considered descriptively, subgroup analyses were not performed due to limited sample size (Table 4) this revealed that knee flexors contributed the largest proportion of explained variance in total isometric strength (β = 0.45), followed by hip flexors (β = 0.30) and plantar flexors (β = 0.20). VIFs ranged between 1.2 and 1.8, and all tolerance values exceeded 0.5, indicating acceptable multicollinearity levels.

## Discussion

The present study’s primary purpose was to provide a profile of the lower limb isometric strength of seven muscle groups during the developmental stages of childhood between 7 and 15 years. The values obtained may contribute to the Chilean isometric muscle strength profile completing previous research done in the upper limb using HHD (Escobar, Munoz & Dominguez, 2017). Also, this report will help to compare possible isometric muscle strength deficits in relation to a standardized profile of typically developing boys and girls already done for the upper limb (Mendez-Rebolledo et al., 2022). Other data revealed that: (a) from 11 years of age, boys begin to present greater isometric muscle strength than girls in total isometric strength of hip flexors, also for plantar flexors at 14 years of age; (b) in boys, 4 years was the narrow range of progressive development of isometric muscle strength with significant difference, starting at 9 years of age in knee flexors and for girls at 9 years of age, but with a narrow significance difference of 3 years. Finally, the smallest significant difference was of one year in females from 8 to 9 years of age in the muscle group of hip extensors; and (c) the isometric muscle strength of the hip flexors and dorsiflexors explain the highest performance of the total isometric muscle strength of the lower limb.

**Table 2:** Body Mass, Height, Z-score BMI, PAQ-A/C group by age. Values are represented as mean ± standard deviation, Females (F), Males (M). Kilograms (kg), Centimeters (cm).

**Variable**	**Sex**	**Age (yr)**								
		7 (*n* = 32)	8 (*n* = 31)	9 (*n* = 33)	10 (*n* = 36)	11 (*n* = 33)	12 (*n* = 30)	13 (*n* = 33)	14 (*n* = 35)	15 (*n* = 39)
**Participants (n)**	F	14	17	18	16	21	16	17	14	19
	M	18	14	15	20	12	14	16	21	20
**Body Mass (kg)**	F	31.6 ± 11.2	32.3 ± 7.6	44.2 ± 11.7	43.0 ± 11.8	48.0 ± 9.8	48.2 ± 13.0	56.6 ± 7.2	58.0 ± 8.0	55.0 ± 10.5
	M	31. 7 ± 6.7	32.4 ± 7.1	43.0 ± 11.5	42.5 ± 9.5	48.3 ± 10.7	51.9 ± 11.8	56.7 ± 6.6	63.0 ± 13.9	73.9 ± 19.8
**Height (cm)**	F	126.7 ± 7.8	129.5 ± 6.1	142.9 ± 4.2	142,2 ± 7,7	149,2 ± 8,7	153,8 ± 7,2	162,1 ± 4,1	160,6 ± 6,7	161,4 ± 6,4
	M	128.6 ± 6.8	129.7 ± 8.7	139.8 ± 9.9	143,4 ± 8,0	147,6 ± 7,2	156,5 ± 9,4	163,5 ± 5,2	168,1 ± 5,5	172,3 ± 5,4
**Z-score BMI**	F	0.75 ± 1.4	1.03 ± 1.51	1.65 ± 1.11	2.0 ± 1.97	1.73 ± 1.82	0.52 ± 2.18	0.75 ± 0.84	0.61 ± 1.0	0.19 ± 1.14
	M	1.45 ± 1.12	1.54 ± 1.07	1.89 ± 1.28	0.52 ± 1.2	0.6 ± 1.37	−0.04 ± 1.47	0.29 ± 0.71	1.09 ± 1.83	1.31 ± 0.85
**PAQ - A/C**	F	2,4 ± 0,8	2,8 ± 1,0	2,7 ± 0,7	2,7 ± 0,9	2,8 ± 0,8	2,3 ± 0,6	2,4 ± 0,8	2,6 ± 1,2	2,5 ± 0,9
	M	2,4 ± 0,8	2,7 ± 0,9	2,6 ± 1,1	2,9 ± 0,6	2,8 ± 0,9	2,5 ± 1,2	2,9 ± 1,2	2,9 ± 1,2	2,6 ± 0,8

**Table 3:** Means and standard deviation of the maximal isometric muscle strength normalized to body mass in N/kg for all muscle groups of both sexes (*n* =302). Values are presented as mean ± standard deviation. Females (F), Males (M) Superscript letters indicate significant differences (Bonferroni-corrected, *p* < 0.05) between age groups within each sex and muscle group.

**Muscle group**	**Sex**	**Age (yr)**								
		7	8	9	10	11	12	13	14	15
**Hip flexors**	F	2.79 ± 0.89	2.74 ± 0.52	2.24 ± 0.79	2.63 ± 0.98	2.59 ± 0.77	2.86 ± 0.32	2.37 ± 0.24	2.67 ± 0.75	2.58 ± 0.68
	M	2.49 ± 1.0	2.62 ± 0.79^a^	2.44 ± 0.81^b^	2.39 ± 0.77	3.22 ± 1.04	3.15 ± 0.85	2.85 ± 0.39	3.17 ± 1.17	3.38 ± 0.46
	ICC	0.80	0.82	0.83	0.85	0.87	0.88	0.89	0.90	0.91
	SEM	0.08	0.08	0.07	0.07	0.07	0.06	0.06	0.06	0.06
**Hip extensors**	F	2.46 ± 0.39	3.27 ± 0.72	2.33 ± 0.49	2.90 ± 0.84	2.56 ± 0.51	2.59 ± 0.85	2.53 ± 0.42	2.84 ± 0.87	2.62 ± 0.76
	M	2.77 ± 0.89	3.22 ± 0.70	2.83 ± 1.29	2.90 ± 1.02	2.99 ± 0.83	2.84 ± 0.74	3.29 ± 0.46	3.10 ± 0.86	3.06 ± 0.93
	ICC	0.82	0.84	0.85	0.87	0.89	0.90	0.91	0.92	0.93
	SEM	0.07	0.07	0.06	0.06	0.06	0.06	0.06	0.06	0.06
**Hip abductors**	F	2.21 ± 0.43	2.54 ± 0.72	2.37 ± 0.56	2.61 ± 0.70	2.65 ± 0.61	2.70 ± 0.53	2.49 ± 0.22	2.29 ± 0.57	2.49 ± 0.47
	M	2.43 ± 0.63	2.78 ± 0.41	2.60 ± 0.85	2.80 ± 0.57	3.01 ± 0.82	2.90 ± 0.57	2.76 ± 0.58	2.68 ± 0.59	3.14 ± 0.78
	ICC	0.79	0.81	0.82	0.84	0.86	0.87	0.88	0.89	0.90
	SEM	0.08	0.08	0.07	0.07	0.07	0.06	0.06	0.06	0.06
**Knee flexors**	F	2.79 ± 0.89	2.12 ± 0.49	1.90 ± 0.75	2.34 ± 0.44	2.72 ± 0.83	2.71 ± 0.99	2.45 ± 0.30	2.33 ± 0.63	2.79 ± 0.57
	M	2.49 ± 1.00	2.51 ± 0.64	1.94 ± 0.83^a^	2.45 ± 0.91	2.50 ± 0.57^b^	3.06 ± 1.023^c^	2.55 ± 0.59	2.81 ± 0.74	2.56 ± 0.61
	ICC	0.78	0.80	0.81	0.83	0.85	0.86	0.87	0.88	0.89
	SEM	0.08	0.08	0.07	0.07	0.07	0.06	0.06	0.06	0.06
**Knee extensors**	F	2.46 ± 0.39	2.51 ± 0.51	2.56 ± 0.83	3.29 ± 0.59	3.16 ± 0.53	2.53 ± 0.63	2.61 ± 0.67	2.73 ± 0.98	3.19 ± 1.11
	M	2.77 ± 0.89	2.98 ± 0.53	2.59 ± 0.77	3.25 ± 1.27	3.34 ± 1.00	2.61 ± 0.49	2.83 ± 0.83	3.20 ± 0.68	3.40 ± 1.16
	ICC	0.83	0.85	0.86	0.88	0.90	0.91	0.92	0.93	0.94
	SEM	0.07	0.07	0.06	0.06	0.06	0.05	0.06	0.06	0.06
**Plantarflexors**	F	2.21 ± 0.6	2.20 ± 0.60	2.14 ± 0.52	1.73 ± 0.38	2.11 ± 0.40	2.26 ± 0.49	2.21 ± 0.51	1.79 ± 0.34	2.18 ± 0.53
	M	2.03 ± 0.39	2.32 ± 0.34	2.16 ± 0.66	2.22 ± 0.68	2.68 ± 0.63	2.60 ± 0.73	2.70 ± 0.73	2.73 ± 0.59	2.84 ± 0.41
	ICC	0.77	0.79	0.80	0.82	0.84	0.85	0.86	0.87	0.88
	SEM	0.08	0.08	0.07	0.07	0.07	0.06	0.06	0.06	0.06
**Dorsiflexors**	F	1.89 ± 0.51	1.81 ± 0.39	1.65 ± 0.50	1.63 ± 0.33	1.76 ± 0.35	1.93 ± 0.54	1.84 ± 0.44	1.76 ± 0.69	1.77 ± 0.57
	M	1.70 ± 0.45	1.75 ± 0.53	1.64 ± 0.44	1.70 ± 0.40	2.00 ± 0.72	2.12 ± 0.54	1.97 ± 0.56	2.24 ± 0.61	2.29 ± 0.53
	ICC	0.76	0.78	0.79	0.81	0.83	0.84	0.85	0.86	0.87
	SEM	0.08	0.08	0.07	0.07	0.07	0.06	0.06	0.06	0.06

**Table 4:** Multiple linear regression model of the total muscle strength of lower limb adjusted by the addition of factors (independent variables).

**Variable**	***R*** ^2^	Δ***R***^2^	β	**SE**	**95% CI**		***P*** **-value**
Hip flexors	0.621	0.621	0.788	0.199	3.213	3.997	0.0001
Dorsi flexors	0.747	0.126	0.453	0.293	2.355	3.512	0.0001
Hip extensors	0.823	0.076	0.324	0.153	1.110	1.712	0.0001
Knee extensors	0.897	0.073	0.325	0.112	1.111	1.555	0.0001
Hip abductors	0.950	0.053	0.273	0.100	1.252	1.647	0.0001
Plantar flexors	0.972	0.023	0.193	0.084	0.898	1.229	0.0001
Knee flexors	1.000	0.028	0.203	0.001	0.996	1.001	0.0001

**Notes.**

SE, standard error, CI, confidence interval.

Δ*R*^2^* represents the percentage of variability (change) that explains the consecutive addition of each of the independent variables to the regression model.

Research in isometric strength in children has considered various populations, ethnicities and clinical conditions (Daloia et al., 2018; Hébert et al., 2015; Mulder-Brouwer, Rameckers & Bastiaenen, 2016). In muscle strength of lower limb studies, the focus has been on comparing it with physiological parameters, chronic disease or birth conditions (Mendez-Rebolledo et al., 2023; Saarinen et al., 2008; Saarinen et al., 2008). For example, performance on the 6-minute walk test—a widely recognized field-based measure of locomotor capacity due to its simplicity, affordability, and established validity in pediatric populations—was significantly associated with isometric hip flexor strength, which accounted for approximately 50% of the variance in walking distance among children and adolescents aged 7 to 15 years (Mendez-Rebolledo et al., 2023). In the present study, more than half of the total maximum isometric muscle strength of the lower limb is attributed to the hip flexor muscle group, considering age as a relevant factor when it is below 10 years of age. Previous studies show that there are no differences in this muscle group before the age of 10 years (Macfarlane, Larson & Stiller, 2008). Development of muscle strength throughout childhood and adolescence depends on factors like development of lean and fat mass (Almeida-Neto et al., 2021), central nervous system (Hedman et al., 2012), and gonadal maturation (Soliman et al., 2014). All these factors are strongly influenced by environment and genetic expression (Malina & Mueller, 1981; Soliman et al., 2014). One of the objectives was to identify chronological age intervals in which significant gains in isometric muscle strength occur, not to define maturational stages. Based on the Bonferroni-corrected post hoc results, these ‘periods of significant isometric strength progression’ were operationalized as those showing statistically significant differences between consecutive or near-consecutive age groups. While this approach cannot substitute for biological maturity data, it provides clinically useful reference points for isometric strength development using normative isometric profiles. Most of these changes occur in preadolescent and adolescent, which could explain why the multiple comparisons showed significant differences between the ages of 9 and 12 years in age and sex. It is why our findings in isometric muscle strength and those of Escobar, Munoz & Dominguez (2017) on eccentric muscle strength, show that in average of 11 years of ages changes between sexes are observed, apparently influenced by bone length (height) and body mass. There is also a marked interaction between boys and girls in plantar flexor muscles that secondly contributes to all around isometric strength of the lower limb. This finding could relate to children that lose lower limb strength with neuromuscular disorders lose up to 73% of plantar flexor force (Stackhouse, Binder-Macleod & Lee, 2005)*,* and the relation between selective activation with other muscles groups like knee extensors (Suzuki, Chino & Fukashiro, 2014). Nonetheless, the differences reported in this study could be due to variation in the methodologies used to calculate and report the results, nonetheless in this case normalization of the data was done body mass (Daloia et al., 2018; Escobar, Munoz & Dominguez, 2017; Van den Beld et al., 2006) or it can be also related to assessed methods, such as stabilization belts (Eek & Lidman, 2021; Hébert et al., 2011) or the use of the break test.

For what is known the present research is the only study that has assessed isometric muscle strength of the lower limb, considering the different muscle groups that contribute to the overall strength. Previous studies have assessed some muscle groups of the lower limb and their role in the total body strength (Escobar, Munoz & Dominguez, 2017; Hébert et al., 2015). In our research we identify that hip flexors, dorsiflexors, hip extensors and knee extensors explained the highest percentage of variance (*R*^2^ = 0.897). The hip flexors were the group that contributed the greatest proportion (chance in *R*^2^ = 0.621) which could be considered the target group to be prospectively assessed in development of the total muscle strength lower limb muscle strength in girls and boys. This could establish a profile for the Chilean schoolchildren population enabling the definition of normal parameters for the control and monitoring of the school-aged population.

The limitations that we have encountered in the present study were the sample which was selected from the convenience of a population of schools. Although our total sample was adequate, the number of participants per age and sex group was relatively modest. However, this distribution is comparable to previous HHD-based studies conducted in different parts of the world, providing useful normative references. We acknowledge that this may limit the generalizability of certain subgroup comparisons and recommend that future studies consider larger or longitudinal samples for greater statistical power and population representativeness. This did not allow the data of the isometric muscle strength to be considered as reference values, however the schools considered were private/subsidized, which in Chile is a category of educational establishments that is representative of a specific social-economic and cultural population group. Also, the lack of control over food intake and failure to complete a food record (to ascertain participants’ dietary profiles, including their intake of protein, carbohydrates, lipids, and micronutrients). It is important to note and to interpret the results of this study, that the absences of biological maturation, which were not assessed in this study, may have influenced the variability in muscle strength observed among participants within the same age groups. In addition, relying solely on chronological age groups may obscure substantial inter-individual variability in biological maturity. Children of the same age may differ markedly in maturation timing, neuromuscular development, and lean mass accrual, which are known to influence strength performance independently of age (Malina, Bouchard & Bar-Or, 2004; Lloyd & Oliver, 2012). Therefore, part of the variability within age groups likely reflects differing maturity status rather than true developmental differences. Previous research suggests that youth closer to or beyond their peak height velocity exhibit greater muscular strength, independent of chronological age. The age- and sex-related differences observed in isometric strength are likely underpinned by multiple physiological mechanisms. Prepubertal children exhibit lower voluntary muscle activation due to incomplete maturation of the central nervous system, particularly in cortical motor areas, which limits maximal motor unit recruitment (Dotan et al., 2012). During adolescence, hormonal changes—especially increased testosterone in boys—stimulate muscle hypertrophy and enhance neuromuscular coordination (Malina, Bouchard & Bar-Or, 2004). Additionally, improvements in motor unit synchronization, firing frequency, and neural drive contribute to greater force generation (Ramsay et al., 1990). These maturational processes align with the marked strength gains observed from age 10 and the greater strength levels seen in males by age 15 in our data.

While absolute agreement metrics such as Bland–Altman plots were not included, recent evidence suggests that SEM offers comparable interpretive value in group-level isometric strength assessments using HHD (Jorquera-Cáceres et al., 2024). Our low SEM values further support the reliability of our measurements within the context of normative pediatric data collection.

Our analyses revealed discrepancies between results obtained with absolute and weight-normalized strength values. While absolute values (N) reflect the total force produced, they are strongly influenced by body size, which complicates comparisons between children of different ages and sexes. For this reason, we normalized strength by body weight (N/kg), an approach widely used in pediatric dynamometry research (Hébert et al., 2011; Daloia et al., 2018; Escobar, Munoz & Dominguez, 2017). This method reduces the confounding effect of body size and enables a more meaningful evaluation of relative strength capacity, which is functionally more relevant in clinical and developmental contexts. Nonetheless, weight normalization assumes a linear strength-to-mass ratio, which may not reflect maturational changes. Alternative metrics, such as lean body mass or muscle quality (force per unit of active muscle tissue), could provide greater physiological precision and should be incorporated in future studies.

An additional limitation of the study is the use of body mass for isometric strength normalization, which may not reflect actual muscle contractile capacity, particularly in participants with higher fat mass or more advanced pubertal development. Evidence suggests that lean body mass and biological maturity are better predictors of strength performance than body weight alone (Almeida-Neto et al., 2021; Yapici et al., 2022). Future studies should incorporate direct assessments of lean mass, such as a dual-energy X-ray absorptiometry scan (DXA) or bioelectrical impedance analysis (BIA), to allow more physiologically meaningful normalization of isometric strength. Such approaches may refine interpretation of strength capacity by distinguishing contractile from non-contractile tissue and improving developmental comparisons across youth populations.

We addressed a key methodological limitation by replacing raw BMI values with age- and sex-adjusted BMI-for-age Z-scores, calculated using WHO growth references. This allowed for a more valid comparison of nutritional status across a heterogeneous pediatric sample. Nonetheless, unmeasured variation in body composition (*e.g.*, fat *vs.* lean mass) may still influence strength outcomes and should be further explored in future studies. Although the cross-sectional design limits the analysis of individual strength progression over time, future studies should incorporate biological maturity indicators and evaluate the rate and acceleration of strength gain to better understand developmental trajectories.

## Conclusion

In summary, our findings suggest that the isometric strength profile of lower limb muscles in this sample of Chilean schoolchildren and adolescents (aged 7–15 years) shows a progression that becomes more evident from age 10, particularly in ventral and distal muscle groups. Sex-related differences were detected in some muscle groups and at specific ages, but these differences were not consistent across all ages or variables. In addition, hip flexors contributed most strongly to total lower limb strength, highlighting their role as key determinants of overall performance. These findings should be interpreted with caution given the modest subsample sizes and the cross-sectional design, and they should not be generalized to the broader Chilean population. Future longitudinal studies including biological maturity assessments are needed to confirm and extend these results. Nevertheless, the present study provides preliminary reference information that may help clinicians and researchers compare strength profiles within and between individuals, and to identify potential deficits, particularly in bilateral impairments.

##  Supplemental Information

10.7717/peerj.20799/supp-1 Supplemental Information 1 STROBE Checklist

10.7717/peerj.20799/supp-2 Supplemental Information 2 Z-score BMI

10.7717/peerj.20799/supp-3 Supplemental Information 3 Raw Data of Muscle Strength The raw measurements of the strength measured by muscle group.

## Additional Information and Declarations

### Data Availability

The following information was supplied regarding data availability: The raw data is available in the Supplemental File.
