# Supplementary material for: Isometric muscle strength profile of lower limbs for children and adolescents between 7 and 15 years of age
Source: PeerJ. 2026 Feb 23;14:e20799. doi: 10.7717/peerj.20799 (PMC12939793; doi:10.7717/peerj.20799)
Supplement: Supplemental Information 1 [file peerj-14-20799-s001.docx]

STROBE Statement—checklist of items that should be included in reports of observational studies

|  | Item No. | Recommendation | Page  No. | Relevant text from manuscript |
| --- | --- | --- | --- | --- |
| **Title and abstract** | 1 | (*a*) Indicate the study’s design with a commonly used term in the title or the abstract | Pag. 2 | cross-sectional study |
|  |  | (*b*) Provide in the abstract an informative and balanced summary of what was done and what was found | Pag.2 |  |
| Introduction | | | |  |
| Background/rationale | 2 | Explain the scientific background and rationale for the investigation being reported | Pag.3 – 5 |  |
| Objectives | 3 | State specific objectives, including any prespecified hypotheses | Pag. 5 |  |
| Methods | | | |  |
| Study design | 4 | Present key elements of study design early in the paper | Pag. 5 |  |
| Setting | 5 | Describe the setting, locations, and relevant dates, including periods of recruitment, exposure, follow-up, and data collection | Pag. 6 | Children were recruited geographically from local schools of the central area of Chile, between the ages of 7 to 15 years |
| Participants | 6 | (*a*) *Cohort study*—Give the eligibility criteria, and the sources and methods of selection of participants. Describe methods of follow-up  *Case-control study*—Give the eligibility criteria, and the sources and methods of case ascertainment and control selection. Give the rationale for the choice of cases and controls  *Cross-sectional study*—Give the eligibility criteria, and the sources and methods of selection of participants | Pag. 6 | Inclusion criteria for participants demanded intact cognitive functions to understand the orders given by the evaluator. Exclusion of the participants were if they presented (a) pain during assessment and procedures, (b) a history of medical, neurological, or musculoskeletal impairments that could affect muscle strength measurements, (c) previous surgeries of the lower limbs and/or spine, or participation in competitive sports during or six months prior to the measurements |
|  |  | (*b*) *Cohort study*—For matched studies, give matching criteria and number of exposed and unexposed  *Case-control study*—For matched studies, give matching criteria and the number of controls per case |  |  |
| Variables | 7 | Clearly define all outcomes, exposures, predictors, potential confounders, and effect modifiers. Give diagnostic criteria, if applicable | Pag 6 – 7 |  |
| Data sources/ measurement | 8* | For each variable of interest, give sources of data and details of methods of assessment (measurement). Describe comparability of assessment methods if there is more than one group | Pag. 6 – 7 |  |
| Bias | 9 | Describe any efforts to address potential sources of bias | - |  |
| Study size | 10 | Explain how the study size was arrived at | Pag. 5 – 6 | the largest width of the confidence interval (W) and the largest standard deviation (S) of maximum isometric strength reported in a previous study (Hébert et al., 2015) were used to calculate the sample size.  Where zα´=1.96, S=0.27, and W=0.38. A minimum of seven participants per sex per age group was required. Children were recruited geographically from local schools of the central area of Chile, between the ages of 7 to 15 years. |

Continued on next page

| Quantitative variables | | 11 | | Explain how quantitative variables were handled in the analyses. If applicable, describe which groupings were chosen and why | Pag. 8- 9 |  | | |
| --- | --- | --- | --- | --- | --- | --- | --- | --- |
| Statistical methods | | 12 | | (*a*) Describe all statistical methods, including those used to control for confounding | Pag. 9 | Data were analyzed using IBM SPSS Statistics ver. 25.0 (IBM Co., Armonk, NY, USA). A significance level (alpha level) of <0.05 was used for all tests. Grouped by age and sex, means and standard deviation were calculated for the maximum isometric muscle strength of the lower limb in children and adolescents. Outlays were considered and removed when their values were >3 standard deviations from the mean. Additionally, normality distribution of the data was assessed by the Shapiro-Wilk test (Hébert et al., 2015). | | |
|  |  |  |  | (*b*) Describe any methods used to examine subgroups and interactions | Pag. 9 | To determine the age at which differences in isometric muscle strength were observed between participant´s sex, a two-factor analysis of variance (ANOVA) (age and sex) was performed for the variable maximum isometric strength of each muscle group and the total muscle strength of lower limb. In cases where significant interactions were found, a Benferroni´s post hoc analysis of multiple comparison test was performed. | | |
|  |  |  |  | (*c*) Explain how missing data were addressed | - |  | | |
|  |  |  |  | (*d*) *Cohort study*—If applicable, explain how loss to follow-up was addressed  *Case-control study*—If applicable, explain how matching of cases and controls was addressed  *Cross-sectional study*—If applicable, describe analytical methods taking account of sampling strategy | Pag. 9 – 10 | To determine the age at which differences in isometric muscle strength were observed between participant´s sex, a two-factor analysis of variance (ANOVA) (age and sex) was performed for the variable maximum isometric strength of each muscle group and the total muscle strength of lower limb. In cases where significant interactions were found, a Benferroni´s post hoc analysis of multiple comparison test was performed. To determine the age range at which significant progression of isometric muscle strength could be observed in girls and boys, the narrowest age range of strength was identified by significant differences (P <0.05) observed between the lowest and highest age of the interval selected. To examine the effect size an Eta-squared (η2) for ANOVA was used where less than 0.06 was classified as “small,” 0.07–0.14 as “moderate,” and greater than 0.14 as “large”. Furthermore, Cohen d for paired samples was used as an indicator of the effect size, where less than 0.2 was classified as “trivial,” 0.2–0.5 as “small,” 0.5–0.8 as “moderate”, and greater than 0.8 as “large” (Cohen, 1992).  Finally, to identify the role of the isometric strength of each muscle group on the total muscle strength of lower limb, a relationship between the maximum isometric strength of each muscle group and the total muscle strength of lower limb was analyzed using Pearson correlation test, where a correlation coefficient (r) from 0–0.4 was considered as “weak”, 0.41–0.7 as “moderate”, and 0.71–1.0 as “strong”. A stepwise multiple linear regression analysis was then performed. using as a dependent variable the total lower limb strength. On the other hand, the independent variable was the maximum isometric strength of each muscle group, adjusted for sex, height, and age. For this method, the initial independent variable selected for analysis was the one that showed the strongest, simple, significant correlation with the total muscle strength of lower limb. The remaining variables that showed simple significant correlations (from highest to lowest correlation) were consecutively added to this model. Determining the goodness of fit was done by means of the R2 coefficient and its percentage of change. Additionally, collinearity diagnoses were performed, being verified through values less than 0.10 tolerance, and identifying the variance inflation factor (VIF), which leds to eliminate the variables that showed collinearity with a VIF >10, in order to define the definitive multiple linear regression model. | | |
|  |  |  |  | (*e*) Describe any sensitivity analyses | - |  | | |
| Results | | | | | | | | |
| Participants | | 13* | | (a) Report numbers of individuals at each stage of study—eg numbers potentially eligible, examined for eligibility, confirmed eligible, included in the study, completing follow-up, and analysed | Pag. 10 | A total of 302 individuals (50,3% female) were included in the analysis | | |
|  |  |  |  | (b) Give reasons for non-participation at each stage | - |  | | |
|  |  |  |  | (c) Consider use of a flow diagram | - |  | | |
| Descriptive data | | 14* | | (a) Give characteristics of study participants (eg demographic, clinical, social) and information on exposures and potential confounders | Pag. 19 | Figure 1 | | |
|  |  |  |  | (b) Indicate number of participants with missing data for each variable of interest | - |  | | |
|  |  |  |  | (c) *Cohort study*—Summarise follow-up time (eg, average and total amount) |  |  | | |
| Outcome data | | 15* | | *Cohort study*—Report numbers of outcome events or summary measures over time |  |  | | |
|  |  |  |  | *Case-control study—*Report numbers in each exposure category, or summary measures of exposure |  |  | | |
|  |  |  |  | *Cross-sectional study—*Report numbers of outcome events or summary measures | *Pag. 20 – 21* | *Table 3* | | |
| Main results | | 16 | | (*a*) Give unadjusted estimates and, if applicable, confounder-adjusted estimates and their precision (eg, 95% confidence interval). Make clear which confounders were adjusted for and why they were included |  |  | | |
|  |  |  |  | (*b*) Report category boundaries when continuous variables were categorized | Pag. 6 | Age of participants | | |
|  |  |  |  | (*c*) If relevant, consider translating estimates of relative risk into absolute risk for a meaningful time period |  |  | | |
| Continued on next page Other analyses | 17 | | Report other analyses done—eg analyses of subgroups and interactions, and sensitivity analyses | |  | |  |  |
| Discussion | | | | | | | |  |
| Key results | 18 | | Summarise key results with reference to study objectives | | Pag. 2 | | The narrowest age range in the progression of maximum isometric strength were: 9-11 years for knee flexors in females (P = 0,0201) and 9-12 years for males (P = 0,0008). Hip flexors, dorsiflexors, hip extensors and knee extensors explained the highest percentage of variance (R² = 0.897, P < 0.0001) in the total lower limb strength |  |
| Limitations | 19 | | Discuss limitations of the study, taking into account sources of potential bias or imprecision. Discuss both direction and magnitude of any potential bias | | Pag. 14 – 15 | | The limitation that we have encounter in the preset study were (a) the sample was selected from the convenience of a population of schools, (b) this did not allow the data of the isometric muscle strength to be considered as reference values, however the schools considered were private/subsidized, which in Chile is a category of educational establishments that is representative of a specific social-economic and cultural population group. Also, the lack of control over food intake and failure to complete a food record (to ascertain participants´ dietary profiles, including their intake of protein, carbohydrates, lipids, and micronutrients). |  |
| Interpretation | 20 | | Give a cautious overall interpretation of results considering objectives, limitations, multiplicity of analyses, results from similar studies, and other relevant evidence | | Pag.15 | | the maximum isometric muscle strength of the lower limb in Chilean children and adolescents between 7 and 15 years of age. This study identified that the most progressive isometric muscle strength development occurs from the age of 10 years approximately, in extensors and flexors groups of muscle of the lower limb, this difference consolidates the age of 15 years between girls and boys. In addition, the total isometric muscle strength of the lower limb is explained mainly by hip flexors, that explain the higher total muscle strength of lower limb performance. |  |
| Generalisability | 21 | | Discuss the generalisability (external validity) of the study results | | Pag. 15 | | profiles maximum isometric muscle strength in Chilean children aged 7–15, highlighting significant development from age 10, gender differences by 15, and hip flexors' key role in overall lower limb strength |  |
| Other information | | |  | | | | |  |
| Funding | 22 | | Give the source of funding and the role of the funders for the present study and, if applicable, for the original study on which the present article is based | | Pag. 15 | | This work was supported by the Internal Contest of Research Projects 2021-2022 (Code 11500032) of the General Directorate of Applied Research and Innovation (DGIAi) of the Universidad Santo Tomas, Chile |  |

*Give information separately for cases and controls in case-control studies and, if applicable, for exposed and unexposed groups in cohort and cross-sectional studies.

**Note:** An Explanation and Elaboration article discusses each checklist item and gives methodological background and published examples of transparent reporting. The STROBE checklist is best used in conjunction with this article (freely available on the Web sites of PLoS Medicine at http://www.plosmedicine.org/, Annals of Internal Medicine at http://www.annals.org/, and Epidemiology at http://www.epidem.com/). Information on the STROBE Initiative is available at www.strobe-statement.org.
